# Interleukin-10-engineered mesenchymal stem/stromal cells exhibit robust immunomodulatory effects in vitro and in vivo

**DOI:** 10.1186/s13287-026-05093-3

**Published:** 2026-06-17

**Authors:** Diego de Carvalho Carneiro, Cássio Santana Meira, Rosane Borges Dias, Vinícius Pinto Costa Rocha, Patrícia Kauanna Fonseca Damasceno, Josiane Dantas Viana Barbosa, Milena Botelho Pereira Soares

**Affiliations:** 1 Gonçalo Moniz Institute, Oswaldo Cruz Foundation (FIOCRUZ), Salvador, Bahia Brazil; 2 SENAI Institute of Innovation in Advanced Health Systems, University SENAI CIMATEC, Salvador, Bahia Brazil; 3 Department of Biological Sciences, State University of Feira de Santana (UEFS), Feira de Santana, Bahia, Brazil

**Keywords:** Inflammation, Sepsis, Endotoxemia, IL-10, Lentivirus, Gene and cell therapy

## Abstract

**Introduction:**

A dysregulated inflammatory response to infection can lead to sepsis, a leading cause of mortality worldwide, and effective anti-inflammatory therapies remain limited. Mesenchymal stem/stromal cells (MSCs) are attractive candidates as immunomodulatory agents. This study evaluated whether genetic modification of MSCs to express interleukin-10 (IL-10), a key anti-inflammatory cytokine, enhances their immunomodulatory effects.

**Methods:**

Bone marrow-derived MSCs from C57Bl/6 mice were genetically engineered by lentiviral transduction to express mouse IL-10 (MSC-IL-10). The immunomodulatory activity in vitro was assessed by co-cultures with macrophages stimulated with LPS and IFN-γ, as well as in Con A–stimulated splenocytes. BALB/c mice subjected to lipopolysaccharide (LPS)-induced endotoxemia were treated with vehicle, dexamethasone, wild-type MSCs (MSC-WT), or MSC-IL-10. Survival, plasma cytokines, leukocyte profiles, CD11b⁺ inflammatory cells, and organ histopathology and biodistribution were evaluated in vivo.

**Results:**

MSC-IL-10 maintained the mesenchymal phenotype and multipotent characteristics while exhibiting robust IL-10 expression. In in vitro assays, MSC-IL-10 significantly decreased the production of the cytokines TNF-α, IL-1β, IL-6, IL-12 or *Nos2* expression by stimulated macrophages or splenocytes, demonstrating superior immunomodulatory effects compared to MSC-WT. In in vivo mice models, MSC-IL-10 significantly reduced systemic pro-inflammatory cytokines, restored circulating leukocyte counts, and attenuated CD11b⁺ (Mac-1 integrin) inflammatory cell recruitment, surpassing MSC-WT-treated groups. Importantly, MSC-IL-10 mitigated tissue damage mainly to lungs and exhibited biodistribution to liver, lungs and spleen in LPS-challenged mice.

**Conclusions:**

These results support an enhanced immunomodulatory effect of IL-10-expressing MSCs as a promising cell-based therapeutic approach for sepsis and other inflammatory and immune mediated disorders.

**Supplementary Information:**

The online version contains supplementary material available at 10.1186/s13287-026-05093-3.

## Introduction

Inflammation plays a key role in the protection against infections, but also in restoring the body’s homeostasis, as well as repairing, reshaping, and renewing tissues in a range of challenging medical conditions. Infections from Gram-negative bacteria can trigger inflammation by lipopolysaccharides (LPS) activation of toll like receptor 4 (TLR4), which initiates a central inflammatory response mediated by MyD88-dependent and TRIF-dependent intracellular signaling, leading to the activation of the transcription factors NF-κB and IRF3 [1, 20]. Activation of TLR4 results in the production of pro-inflammatory cytokines, chemokines, and vasoactive mediators that promote vasodilation, increased vascular permeability, and the recruitment of leukocytes to the site of infection [5].

When the inflammatory response becomes excessive or poorly regulated, it can culminate in sepsis, which is a complex clinical syndrome characterized by a dysregulated systemic inflammatory response to infection, resulting in potentially fatal organ dysfunction. When sepsis progresses to persistent hypotension and tissue hypoperfusion despite adequate fluid resuscitation, it is defined as septic shock. Endotoxic shock refers specifically to sepsis caused by Gram-negative bacteria, whose cell walls contain LPS, also known as endotoxins [9, 30].

The immune response in sepsis triggers intracellular signaling pathways and production of pro-inflammatory cytokines, especially tumor necrosis factor alpha (TNF-α), interleukin-1 beta (IL-1β), and interleukin-6 (IL-6) [1]. Massive cytokine release causes systemic inflammation, vasodilation, endothelial activation, increased vascular permeability, and leukocyte recruitment, leading to hypotension, tissue injury, and microthrombosis [11, 16]. Microvascular dysfunction and hypotension impair oxygen delivery, resulting in cellular hypoxia, mitochondrial dysfunction, and multi-organ failure, particularly in the lungs, kidneys, liver, and heart. In parallel, excessive generation of reactive oxygen species further amplifies oxidative stress and exacerbates tissue damage, reinforcing the severity of septic pathology [2].

In 2017, an estimated 49 million cases of sepsis occurred worldwide, with 11 million deaths, representing nearly 20% of all global mortality. Sepsis is especially prevalent in low- and middle-income countries and remains a leading cause of preventable death. Hospital mortality rates are still high, around 17% in milder cases and up to 26% in severe cases, with even higher rates in septic shock. Risk groups include elderly people, neonates, immunocompromised individuals (e.g., cancer patients, HIV/AIDS, transplant recipients), and critically ill patients undergoing invasive procedures, long-time hospitalization and/or prolonged antibiotic use [26].

Mesenchymal stem/stromal cells (MSCs) are multipotent, low-immunogenic cells derived from various tissues that exert trophic and immunomodulatory functions, regulating inflammation and promoting tissue repair through the secretion of cytokines such as transforming growth factor-beta (TGF-β), indoleamine (IDO), and prostaglandin E2 (PGE_2_) [32]. These molecules modulate macrophages toward the anti-inflammatory M2 phenotype, inhibit effector T-cells, reduce Th1/Th17-biased immune responses, and promote regulatory T-cells expansion. Their effects are enhanced by “licensing” within inflammatory microenvironments and are also mediated by extracellular vesicles carrying miRNAs and proteins with therapeutic potential [34]. After infusion, MSCs may undergo apoptosis followed by its phagocytosis, which further induces anti-inflammatory responses [21]. Despite a favorable safety profile, challenges remain regarding heterogeneity, lack of conclusive clinical trials, and standardization, while promising strategies include the use of extracellular vesicles, preconditioning, and genetic engineering to optimize clinical applications [23].

Non-steroidal anti-inflammatory drugs or glucocorticoids are traditional treatments for inflammation. These medications vary in their ability to reduce inflammation and can lead to a range of side effects, including atherosclerosis, cardiac diseases, digestive disorders, high blood pressure, kidney toxicity, type 2 diabetes, and visceral obesity. Based on that, there is an urgent need for novel anti-inflammatory therapies that are effective and associated with fewer side effects, which is critical to improving clinical outcomes [1, 11].

Interleukin-10 (IL-10) possesses potent anti-inflammatory, regulatory and regenerative properties by limiting immune responses: suppressing phagocytic activity, inhibiting Th1/Th2 responses, activating regulatory T cells, and reducing pro-inflammatory cytokine production [27]. Therefore, IL-10 has emerged as a promising therapeutic candidate in sepsis research [28]. This study aims to evaluate in vitro and in in vivo experimental models of endotoxemia the immunomodulatory effects of MSCs genetically modified to express IL-10.

## Materials and methods

### Ethical considerations

This study was conducted in accordance with the ethical principles for animal research established by the Brazilian Law and was approved by the Animal Use Ethics Committee from the Gonçalo Moniz Institute, Oswaldo Cruz Foundation (Fiocruz-Bahia). This work was reported in accordance with the ARRIVE 2.0 guidelines (Supplementary Material 2).

### Isolation, culture and characterization of MSCs

C57Bl/6 mice, aged 6 to 8 weeks, were used for the isolation of MSCs from mouse bone marrow, following an adapted protocol as described by Soleimani and Nadri [31] and outlined in Figure S1. Briefly, mice were euthanized by cervical dislocation and aseptically dissected in a laminar flow hood for the removal of the tibiae and femurs from the hind limbs. The bones were washed in saline solution with 1% penicillin-streptomycin (Gibco™, Grand Island, NY, USA), and their epiphyses were cut and inserted cut-side down into 10 µL pipette tips for centrifugation in microtubes at 700 g for 1 min. The collected cells were washed, pelleted and cultured with Dulbecco’s Modified Eagle Medium (DMEM; Gibco™, Grand Island, NY, USA) high glucose culture medium containing 10% fetal bovine serum (FBS; Gibco™, Grand Island, NY, USA) and 1% penicillin-streptomycin in cell culture flasks and maintained in humidified incubators at 37 °C and 5% CO_2_. Passages were performed with trypsin-EDTA (Sigma-Aldrich™, St. Louis, MO, USA), and cells were cryopreserved in FBS with 10% dimethyl sulfoxide (Êxodo^®^, Sumaré, SP, Brazil).

The MSCs were characterized by their ability to adhere to plastic under standard culture conditions after four passages, immunophenotyping by flow cytometry, and trilineage differentiation potential (adipogenic, osteogenic, and chondrogenic). Immunophenotyping criteria included the positive expression of CD105, CD73, and CD29, and the absence of CD45, CD34, and CD11b. Cells were labeled with anti-mouse antibodies (Elabscience^®^, Houston, TX, USA), followed by acquisition of 10,000 events with LRSFortessa flow cytometer (BD Biosciences, Franklin Lakes, NJ, USA). Data analysis was performed with FlowJo software (BD Research Cloud, Ashland, OR, USA). Their potential to differentiate into adipocytes, osteoblasts, and chondroblasts was performed with StemPro^®^ Differentiation Kits (Gibco™, Grand Island, NY, USA), following the manufacturer’s instructions. The cells were fixed with 4% paraformaldehyde (Thermo Scientific™, Waltham, MA, USA), followed by adipogenic staining with Oil Red, osteogenic staining with Alizarin Red S, and chondrogenic staining with Alcian Blue.

### Design, synthesis, and cloning of IL-10 expression vector

The reference CDS sequence of mouse IL-10 (*NM_010548.2*) was retrieved from the National Center for Biotechnology Information (*NCBI*) database. The restriction site for BamHI and the Kozak consensus sequence were inserted at the beginning of IL-10 CDS, while BsrGI was inserted at the end of the sequence. The planned construct was optimized using the GeneArt^®^ platform (Invitrogen™, Carlsbad, CA, USA) by replacing the codons in the sequence with the most frequent codons for the host organism (*Mus musculus*), maintaining the same amino acid sequence. The synthetic DNA encoding the target sequence was synthesized by GeneArt^®^ in the *pMA-RQ* plasmid, which was cloned in chemically competent *Escherichia coli* DH5α cells via heat shock. The pMA-RQ and *pEGIP* plasmids were digested with the restriction enzymes BamHI and BsrGI, and the digestion products were subjected to 1% agarose gel electrophoresis with SYBR Safe DNA Gel Stain^®^ (Invitrogen™, Carlsbad, CA, USA) in TAE buffer. The band from pEGIP expression cassette (without GFP) and the band from pMA-RQ-IL-10 corresponding to the IL-10 insert were excised from the gel, extracted, and purified using the Pure Link Gel Extraction Kit^®^ (Invitrogen™, Carlsbad, CA, USA) according to the manufacturer’s protocol (Figure S2).

The ligation of the pEGIP vector with IL-10 insert was performed with T4 DNA ligase (Thermo Scientific™, Waltham, MA, USA). The resulting pEGIP-IL-10 vector was cloned in chemically competent *E. coli* DH5α cells. The plasmids were extracted and purified using the QIAGEN Plasmid Plus Maxi Kit^®^ (QIAGEN™, Hilden, Germany), following the manufacturer’s protocol. Then, pEGIP-IL-10 was digested with BamHI and BsrGI to confirm presence of the IL-10 insert (Figure S3) and it was sequenced by capillary electrophoresis using the Sanger method with an initiation primer for the T7 promoter. ClustalW multiple alignment was performed between the sequence obtained from the Sanger sequencing and the predicted sequence for pEGIP-IL-10 to confirm ligation, and it showed 100% similarity with the synthetic IL-10 sequence (Figure S4).

### Genetic modification of MSCs

Human Embryonic Kidney (HEK293T) cells cultured in DMEM high glucose medium with 10% FBS, 1% penicillin-streptomycin, and 0.01 M HEPES (Sigma-Aldrich™, St. Louis, MO, USA) were used to produce non-replicating lentiviruses for delivering of pEGIP-IL-10 to MSCs, following a method adapted from Zou et al., [37]. Lipofectamine™ 3000 Reagent (Invitrogen™, Carlsbad, CA, USA) was used to transfect HEK293T cells, following the manufacturer’s protocol. The third-generation lentiviral system plasmids used are described in Table S1. The same reaction volume was used for transfection of either pEGIP-IL-10 or pEGIP-GFP (unmodified).

For lentivirus titration, the supernatant from HEK293T cells transfected with pEGIP-GFP was used to infect 1 × 10^4^ HEK293T cells plated in a 96-well plate after 2x serial dilution in 10% FBS DMEM high glucose and antibiotics. The number of cells expressing GFP in each well was quantified using the High Content Screening (HCS) CellInsight CX7 LZR platform (Thermo Scientific™, Waltham, MA, USA) to calculate the number of transduction units (TU) per mL. MSCs were infected with 1 MOI (multiplicity of infection) of lentiviruses. The MSCs transduced with pEGIP-IL10 were subjected to selection with 2 µg/mL of puromycin, along with a non-transduced control, for selection of cells with stable integration of recombinant DNA, resulting in the MSC-IL-10 cell line. As expected, the MSC-IL-10 cells did not express GFP, as seen in Figure S5. The genetic modification of MSCs is outlined in Figure S6.

### Assessment of cytokine production by activated splenocyte cultures

The in vitro assays of cytokine quantification in macrophage and splenocyte cultures were performed according to the methodology described by Meira et al., [18]. For the assays with splenocytes, BALB/c mice were euthanized by cervical dislocation and dissected for spleen removal under aseptic conditions in a laminar flow hood. The spleen was mechanically dissociated using the plunger of a 10 mL syringe, and splenocytes were resuspended, collected, and plated in DMEM supplemented with 10% FBS and 1% penicillin–streptomycin at 5 × 10⁵ cells per well.

Splenocytes cultured in 48-well plates were stimulated with 5 µg/mL concanavalin A (Con A; Sigma-Aldrich™, St. Louis, MO, USA). Negative controls consisted of untreated cells, with or without stimulation, and the positive control was 10 µM dexamethasone. Stimulated splenocytes were co-cultured with wild type MSCs (MSC-WT) or MSC–IL-10 at ratios of 1:5, 1:10, and 1:20 (MSC:splenocytes). After 48 h, culture supernatants were collected for quantification of IL-10, IL-2, IFN-γ, and TNF-α by sandwich ELISA assays using Mouse DuoSet^®^ ELISA Kits (R&D Systems™, Minneapolis, MN, US), following the manufacturer’s instructions.

### Assessment of cytokine production by activated macrophage cultures

For the assays with macrophages, BALB/c mice were stimulated with an intraperitoneal injection of 3% thioglycolate (Sigma-Aldrich™, St. Louis, MO, USA) for 4 or 5 days. Mice were euthanized by cervical dislocation and handled under aseptic conditions in a laminar flow hood for collection of peritoneal macrophages. Sterile 0.9% saline solution was injected into the intraperitoneal cavity of the mice for collection of macrophages. Cells were centrifuged at 400 g for 5 minutes, resuspended in DMEM medium with 10% FBS and 1% penicillin-streptomycin, and viable macrophages were counted using a hemocytometer with Trypan Blue (Gibco™, Grand Island, NY, USA). A total of 2 × 10^5^ cells/well were plated in 48-well plates, washed after 24 h to remove non-adherent cells, and incubated at 37 °C in a 5% CO_2_ humidified atmosphere.

Macrophages were stimulated with 500 ng/mL of LPS (Sigma-Aldrich™, St. Louis, MO, USA) and 5 ng/mL of interferon-gamma (IFN-γ; Sigma-Aldrich™, St. Louis, MO, USA). Stimulated macrophages were treated with co-cultures of MSC-WT or MSC-IL-10 in ratios of 1:5, 1:10, and 1:20 (MSC:macrophage) or with the conditioned media from 4 × 10^4^ cells/cm^2^ cultures of MSC-WT or MSC-IL-10. Negative controls were macrophages without treatment and LPS + IFN-γ stimulation, while stimulated controls consisted of macrophages with LPS + IFN-γ stimulation. Positive control consisted of stimulated macrophages treated with dexamethasone at a concentration of 10 µM. The supernatants from the co-cultures and controls were used for quantification of the cytokines IL-10, TNFα, IL-1β, IL-6 and IL-12 by ELISA using Mouse DuoSet^®^ ELISA Kits (R&D Systems™, Minneapolis, MN, US). The quantification of nitric oxide (NO) was performed using the Griess method, as described by Green et al., [10]. Then, cells were collected for RNA extraction and Reverse Transcription quantitative Polymerase Chain Reaction (RT-qPCR).

### IL-10 neutralization assay

To confirm the effects of IL-10 produced by the genetically modified MSCs, macrophages stimulated with LPS + IFN-γ were co-cultured in 48-well plates with MSC-IL-10 at a 1:1 ratio after 30 min of stimulation and treated or not with 50 µg/mL of an IL-10–neutralizing antibody (BioLegend™, San Diego, CA, USA). After 6 h, supernatants were collected and used for quantification of cytokines by ELISA.

### RT-qPCR

TRIzol^®^ (Thermo Scientific™, Waltham, MA, USA) was used for cell lysis and RNA isolation, followed by cDNA synthesis using the High-Capacity cDNA Reverse Transcription Kit^®^ (Thermo Scientific™, Waltham, MA, USA) on a Veriti^®^ thermal cycler (Applied Biosystems™, Foster City, CA, USA), according to the manufacturer’s protocols. qPCR reactions were performed in triplicate using SYBR^®^ Green PCR Master Mix (Applied Biosystems™, Foster City, CA, USA) on a QuantStudio 1 Real-Time PCR System (Applied Biosystems™, Foster City, CA, USA). Relative mRNA expression of targeted genes were calculated for *Ipo8* or *Gapdh* reference genes using the primers listed in Table S2.

### LPS-induced endotoxic shock model

Five randomized experimental groups (*n* = 8/group) of male BALB/c mice with 14–18 g of weight were treated with caudal intravenous (i.v.) injections of vehicle (0,9% saline solution), 1 × 10^6^ MSC-WT, 0.25 or 1 × 10^6^ MSC-IL-10, and intraperitoneal (i.p.) injections of dexamethasone (30 mg/kg), 30 min after challenge with a lethal dose of 600 µg of LPS (Sigma-Aldrich™, St. Louis, MO, USA) in saline via intraperitoneal route, as previously described by Meira et al., [18]. Mice were monitored for survival over a 5-day period. The negative control group received only the vehicle used for MSC suspension. Dexamethasone was used as a positive control.

### Analyses of hematological inflammatory markers

Male BALB/c mice (14–16 weeks of age) were randomized into five experimental groups (*n* = 6/group): non-treated animals (naïve), animals treated with 100 µL of caudal i.v. injections of 1 × 10^6^ MSC-WT, 1 × 10^6^ MSC-IL-10, or vehicle (0,9% saline solution), or i.p. injections of dexamethasone (30 mg/kg) 30 min after i.p. injections of 600 µg of LPS. After 6–8 h, mice were injected i.p. with 500 U/kg of heparin and euthanized by cervical dislocation for collection of blood samples from the peritoneal cavity after removing heart and lungs. Individual hematology analyses were performed in the PE-7010VET hematology analyzer (PROKAN^®^, Shenzhen, China), and plasma samples were used for measurement of the cytokines IL-10, TNFα, IL-1β, and IL-6 by ELISA assays using Mouse DuoSet^®^ ELISA Kits (R&D Systems™, Minneapolis, MN, US). A pool of blood samples per group was collected for red blood cell lysis using ACK lysis buffer (Invitrogen™, Carlsbad, CA, USA), followed by acquisition of 100,000 events with LRSFortessa flow cytometer (BD Biosciences, Franklin Lakes, NJ, USA) for analysis of CD11b^+^ cells with anti-mouse antibodies (Elabscience^®^, Houston, TX, USA). Animals in the naive group were not challenged with LPS and did not receive any treatment.

### Histological analysis

Male BALB/c mice (14–16 weeks of age) were randomized into naïve and treated groups (*n* = 6/group) that received i.p. injections of 600 µg of LPS and i.v. injections of 1 × 10^6^ MSC-WT, 1 × 10^6^ MSC-IL-10, or vehicle, or i.p. injections of dexamethasone 30 min later. Animals were euthanized by cervical dislocation and dissected for collection of lungs, heart, liver, and kidney after 6–8 h. Organs were conserved in 10% formaldehyde and processed in paraffin for histological analysis.

Histological sections of 4 μm thickness were obtained and stained with hematoxylin and eosin (H&E). Additionally, liver and kidneys were subjected to periodic acid–Schiff (PAS) staining for evaluation of hydropic degeneration and the presence of hyaline material, respectively. Unblinded histological analyses were conducted by a pathologist using light microscopy at magnifications of 4x, 100x, 200x, and 400x, and representative photomicrographs were captured. Morphological parameters were classified according to the severity of tissue alterations: absent (0), mild (+ 1), moderate (+ 2), and severe (+ 3).

### Biodistribution assay

Approximately 10 mg of the fluorescent dye DiR (Invitrogen™, Carlsbad, CA, USA) was dissolved in 3.3 mL of DMSO to prepare a 3 mM stock solution. This solution was homogenized and stored at room temperature, protected from light with aluminum foil. For cell labeling, 5 µL of the DiR fluorescent reagent was used for each 1 × 10⁶ cells/mL in 500 µL, followed by incubation at 37 °C for 20 min. The labeled cells were pelleted by centrifugation at 1500 rpm for 5 min, the supernatant was discarded, and the cells were resuspended in 0.9% saline solution three times. Male BALB/c mice were divided into groups receiving either i.v. injections of vehicle only, i.v. injections of 0.25 × 10⁶ labeled MSC-IL-10, or i.p. injections of 600 µg of LPS followed by i.v. injections of 0.25 × 10⁶ labeled MSC-IL-10. After 24 h, mice were euthanized by cervical dislocation, and organs were collected for imaging-based analysis of cell biodistribution using excitation at 710 nm and emission at 770 nm with the amiHTX system (Spectral Instruments Imaging, Bruker™, Tucson, AZ, USA).

### Statistical analysis

Data were statistically analyzed by mean and standard deviation (SD) of three to five replicates using GraphPad Prism 8 in independent experiments. Comparisons between two groups of data measured at the same time were analyzed using Student’s *t*-test. Differences among more than two groups or two time points were analyzed by ANOVA. Survival analysis was performed using the log-rank (Mantel–Cox) test.

## Results

### Mesenchymal stem/stromal cell phenotype

**Fig. 1:**
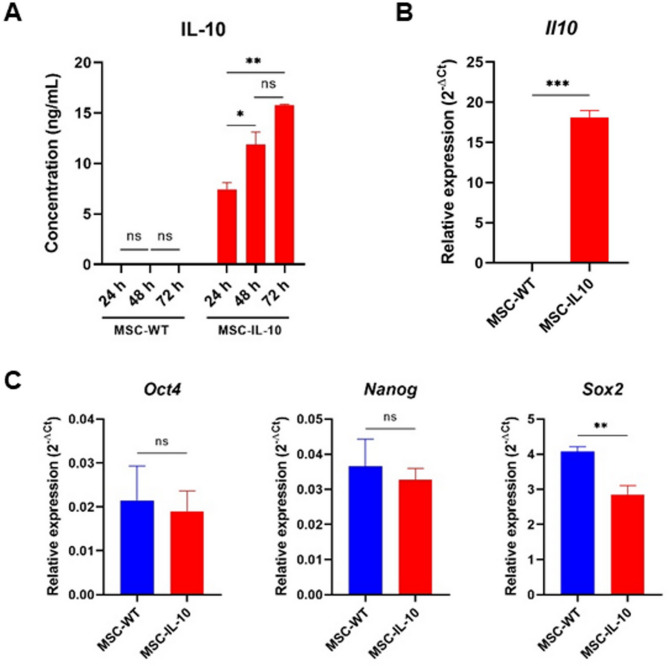
Expression of IL-10 and multipotency genes by the genetically and non-genetically modified MSCs. **A** Production of IL-10 from wild type MSCs (MSC-WT) and IL-10 engineered MSCs (MSC-IL-10) quantified by ELISA in culture supernatants at 24, 48, and 72 h. **B** Optimized IL-10 mRNA expression relative to *Ipo8* from cultured MSC-WT and MSC-IL-10 measured by RT-qPCR. **C** mRNA expression relative to *Ipo8* of the multipotency genes *Oct4*, *Nanog* and *Sox2* from cultured MSC-WT and MSC-IL-10 measured by RT-qPCR. Data are presented as mean ± SD analyzed by *t*-tests or paired two-way ANOVA with Tukey’s test. ns: not significant. *p < 0.05, **p < 0.005, and ***p < 0.0005

IL-10 secretion and expression, multipotency genes expression, trilineage differentiation potential and immunophenotyping were performed to confirm genetic modification and maintenance of MSC phenotype by the IL-10-engineered MSCs. The genetically modified MSCs exhibited a significant increase in secreted IL-10 levels over the 24, 48, and 72-hour periods (Fig. 1A) and mRNA expression of IL-10 (Fig. 1B). In contrast, non–genetically modified MSCs showed no detectable mRNA expression or secretion of IL-10. The concentration of IL-10 secreted into the medium ranged from 6.8 to 15.7 ng/mL over the 72 h period, confirming the ability of MSC-IL-10 to express and secrete IL-10 at high concentrations.

**Fig. 2:**
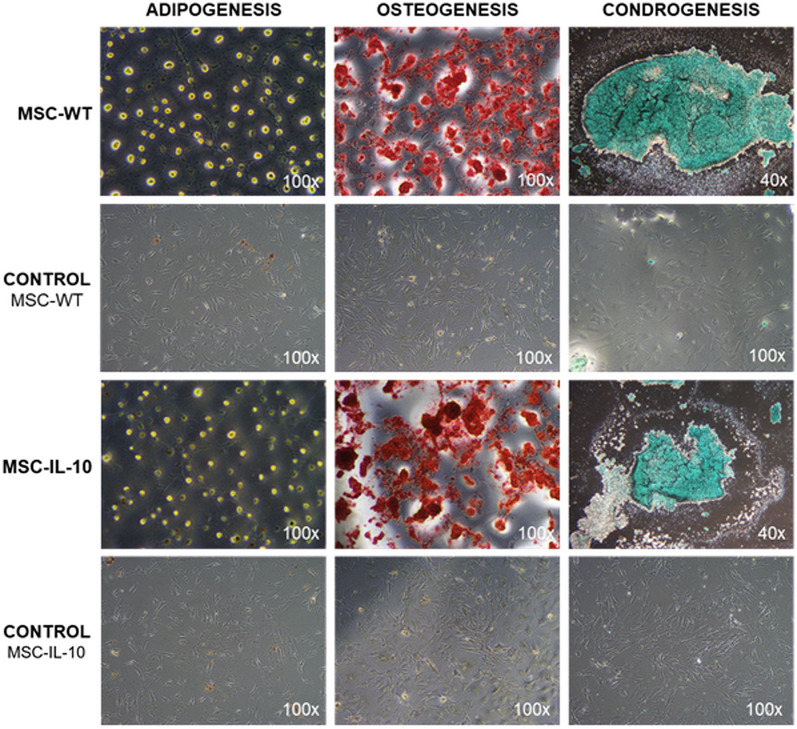
Maintenance of multipotency differentiation potential by the genetically modified MSCs. Adipogenic, osteogenic, and chondrogenic differentiation of wild type MSCs (MSC-WT) and IL-10 engineered MSCs (MSC-IL-10). Cultures were stained with Oil Red (adipogenesis), Alizarin Red S (osteogenesis), or Alcian Blue (chondrogenesis). Controls were cultures without differentiation medium

MSC-WT and MSC-IL-10 demonstrated similar expression levels of the multipotency genes *Oct4* and *Nanog*, while *Sox2* presented a slight but significant reduction in MSC-IL-10 (Fig. 1C). They showed differentiation potential into adipocytes, osteoblasts, and chondroblasts, as demonstrated by staining with Oil Red, Alizarin Red S, and Alcian Blue, respectively, under light microscope (Fig. 2).

**Fig. 3:**
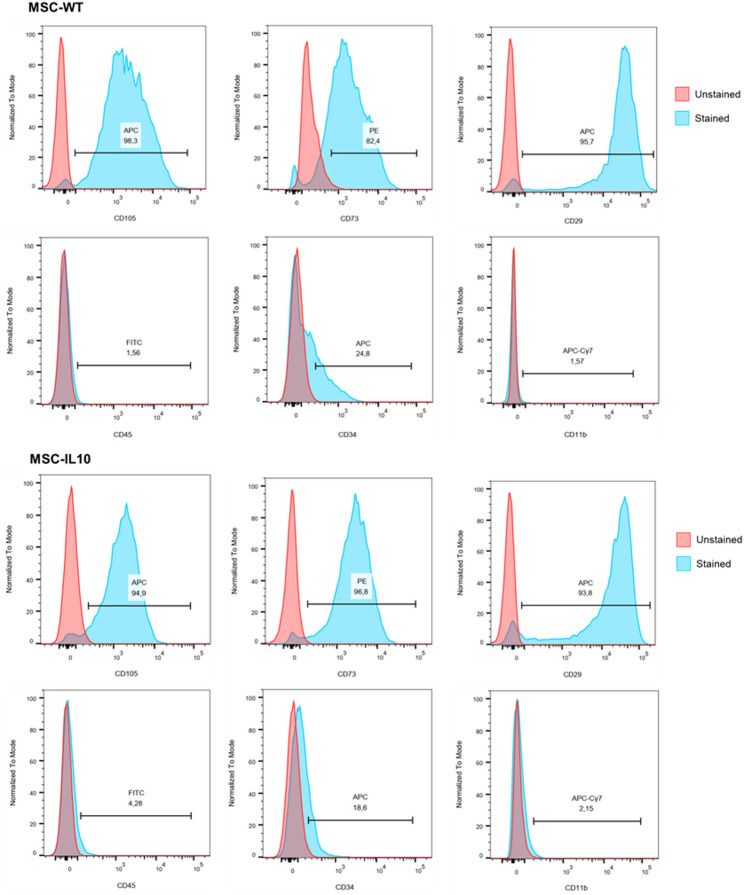
Maintenance of mesenchymal immunophenotype by the genetically modified MSCs. Percentage of cells normalized to mode expressing positive markers (CD105, CD73, and CD29) and negative markers (CD45, CD34, and CD11b) from the cultured wild type MSCs (MSC-WT) and IL-10 engineered MSCs (MSC-IL-10) measured by flow cytometry immunophenotyping. Unstained controls shown by the red peaks are cells not labeled with antibodies. Cells stained with specific fluorescent antibodies for positive and negative markers are shown by the blue peaks

In addition, both MSC-WT and MSC-IL-10 populations exhibited high percentages of cells expressing mesenchymal markers CD105, CD73, and CD29, while showing low expression of hematopoietic markers CD45, CD34, and CD11b (Fig. 3 and Table S3). These results demonstrate that MSC-IL-10 maintained the mesenchymal stem/stromal cell phenotype after genetic modification.

**Fig. 4:**
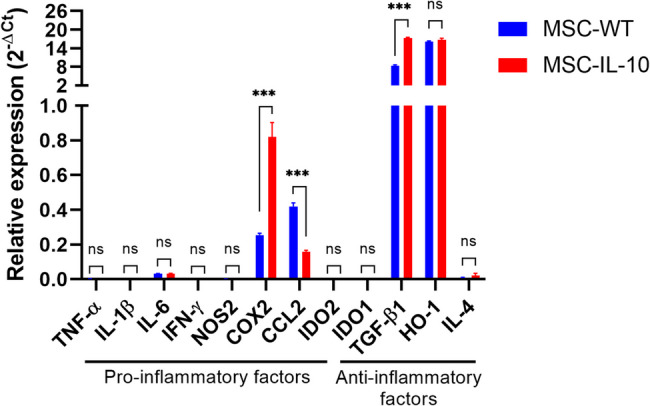
Relative expression of genes coding for pro- and anti-inflammatory factors produced by cultured genetically and non-genetically modified MSCs. The mRNA expression of wild type MSCs (MSC-WT) and IL-10 engineered MSCs (MSC-IL-10) was relative to *Ipo8* as endogenous control for calculation of 2^−ΔCt^. Data are presented as mean ± SD analyzed by *t*-tests. ns: not significant. *p < 0.05, **p < 0.005, and ***p < 0.0005

As seen in Fig. 4, the genetic modification of MSCs to express *Il10* did not change the expression of most immunomodulatory factors potentially expressed by MSCs. However, an increase in the expression of the pro-inflammatory factor *Cox2* and a decrease in *Ccl2* was observed in MSC-IL-10, as well as a significant increase in the expression of the anti-inflammatory factor *Tgfb1*. In addition, both MSC-WT and MSC-IL-10 presented low levels of *Il6* and *Il4* and high levels of *Ho1* expression.

### Immunomodulation in vitro

**Fig. 5:**
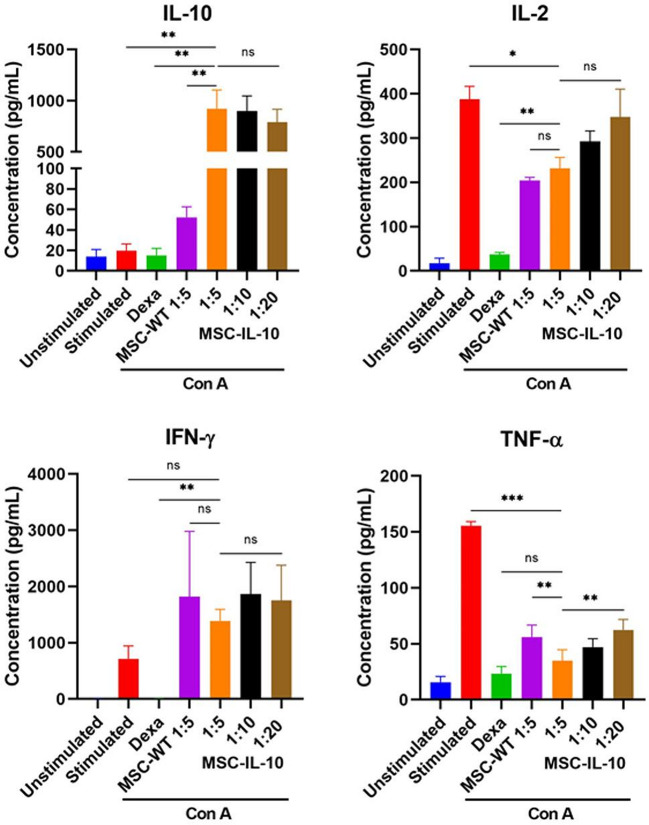
Cytokine concentrations in the supernatant of the in vitro assay with activated splenocyte cultures. Cell-free supernatants were collected 48 h after stimulation for quantification of IL-10, IL-2, IFN-γ, and TNF-α by ELISA. Controls correspond to unstimulated splenocytes (negative) and splenocytes stimulated with Concanavalin A (Con A). Splenocytes were also stimulated with Con A in the treatments with dexamethasone (dexa), wild type MSCs (MSC-WT) and IL-10 engineered MSCs (MSC-IL-10). Co-culture ratios represent the number of MSCs relative to the number of splenocytes (MSC:splenocyte). Data are presented as mean ± SD analyzed by paired one-way ANOVA with Tukey’s test. ns: not significant. *p < 0.05, **p < 0.005, and ***p < 0.0005

Assessments of pro-inflammatory cytokine production by activated splenocytes and macrophages in co-cultures were performed to compare the immunomodulatory effects of IL-10-engineered MSCs with non-genetically modified MSCs and standard dexamethasone treatments. The in vitro cytokine quantification assay performed at 48 h with activated splenocytes co-cultured with MSCs (Fig. 5) showed no difference in IL-2 reduction between wild type MSCs and IL-10-expressing MSCs. Similarly, although treatments with MSCs presented increased IFN-γ production, no significant differences were observed in IFN-γ concentrations in the supernatants of co-cultures with MSC-WT or MSC-IL-10. However, the increase in TNF-α secretion by Con A-stimulated splenocytes showed enhanced mitigation for treatments with MSC-IL-10 compared to MSC-WT, consistent with its IL-10 secretion, and this effect was reduced by dilution of MSC-IL-10. The effects of IL-10-expressing MSCs in splenocytes was similar to that of dexamethasone, a gold standard immunosuppressive agent.

**Fig. 6:**
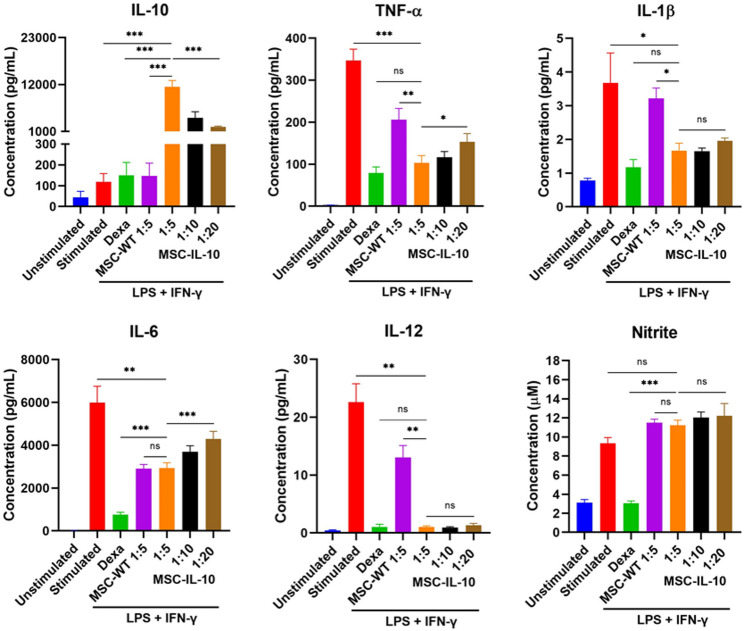
Cytokine concentrations in the supernatant of the in vitro assay with activated macrophage cultures. Supernatants were collected 24 h after stimulation for quantification of IL-10, TNFα, IL-1β, IL-6 and IL-12 by ELISA and nitrite by Griess. Controls correspond to unstimulated macrophages (negative) and macrophages stimulated with LPS + IFN-γ. Macrophages were also stimulated with LPS + IFN-γ in the treatments with dexamethasone (dexa), wild type MSCs (MSC-WT) and IL-10 engineered MSCs (MSC-IL-10). Co-culture ratios represent the number of MSCs relative to the number of macrophages (MSC:MΦ). Data are presented as mean ± SD analyzed by paired one-way ANOVA with Tukey’s test. ns: not significant. *p < 0.05, **p < 0.005, and ***p < 0.0005

In the immunomodulatory activity assay in vitro with activated macrophages (Fig. 6), IL-10-expressing MSCs promoted the reduction of TNF-α, IL-1β, and IL-12 by macrophages stimulated with LPS + IFN-γ, and was significantly more potent than MSC-WT, corresponding to their IL-10 secretion. Importantly, the reduction in TNF-α, IL-1β, and IL-12 observed in the treatment with IL-10-expressing MSCs was not statistically different from those observed with the dexamethasone treatment. Although both MSC cell lines reduced IL-6 production compared to the stimulated control, no significant difference was observed between the MSC-WT and MSC-IL-10 treatments. In addition, neither cell line reduced nitric oxide (NO), as evaluated by the analysis of nitrite concentration in the supernatant using the Griess method.

**Fig. 7:**
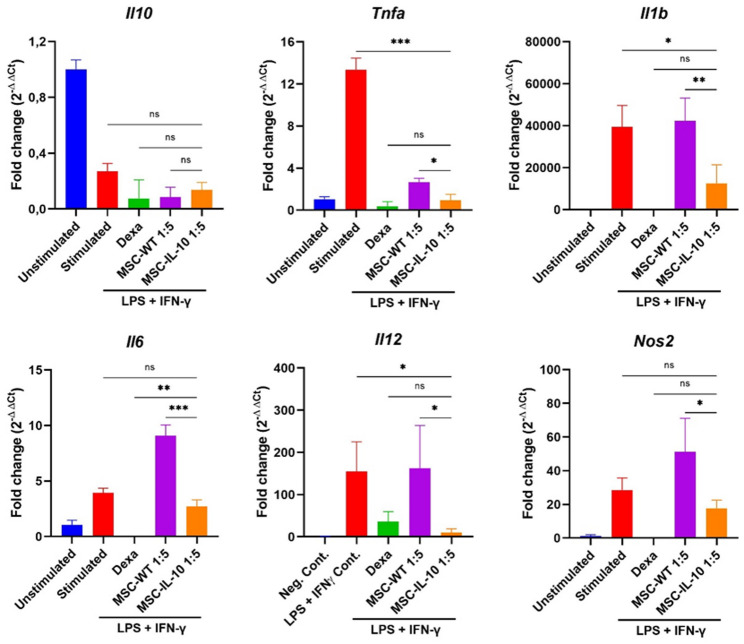
Cytokine gene expression of the in vitro assay with activated macrophage cultures. Controls correspond to unstimulated macrophages (negative) and macrophages stimulated with LPS + IFN-γ. Macrophages were also stimulated with LPS + IFN-γ in the treatments with dexamethasone (dexa), wild type MSCs (MSC-WT) and IL-10 engineered MSCs (MSC-IL-10). The mRNA expression of *Il10*, *Tnfa*, *Il1b*, *Il6*, *Il12* and *Nos2* was relative to *Gapdh* as the endogenous control, and the negative control was used as reference for calculation of 2^−ΔΔCt^ fold-change by RT-qPCR. mRNA extraction was performed 24 h after stimulation. Data are presented as mean ± SD analyzed by paired one-way ANOVA with Tukey’s test. ns: not significant. *p < 0.05, **p < 0.005, and ***p < 0.0005

Similarly, mRNA expression levels of *Tnfa*, *Il1b*, and *Il12* from macrophages were more significantly reduced in MSC-IL-10 than in MSC-WT-treated cultures, relative to the LPS + IFN-γ control. Importantly, the inhibition of cytokine expression promoted by MSC-IL-10 was not significantly different than that observed with dexamethasone treatment, and did not correspond to any *Il10* expression by macrophages, as verified using primers specific for the standard non-optimized IL-10 mRNA (Table S2). In addition, no significant reduction in *Il-6* and *Nos2* expression was observed relative to the LPS + IFN-γ control, despite a greater reduction being observed with MSC-IL-10 compared with MSC-WT (Fig. 7).

**Fig. 8:**
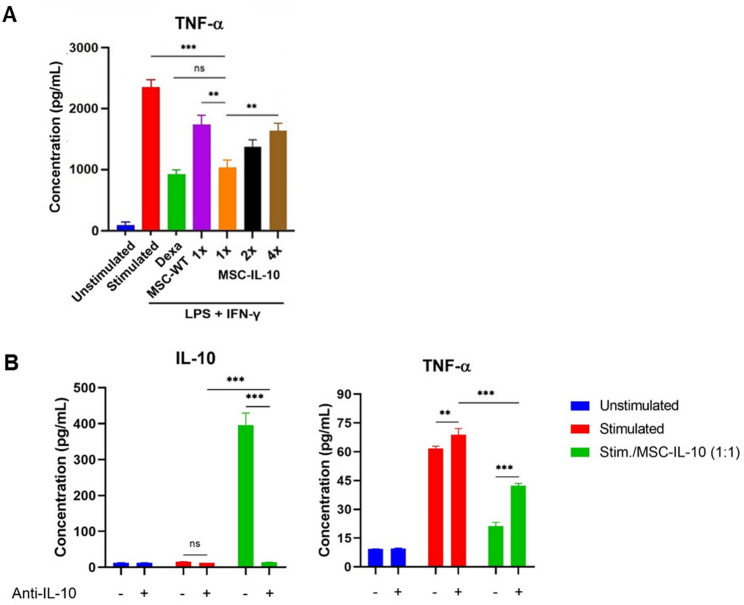
Effects of the MSCs conditioned media and the IL-10 neutralization on TNF-α concentration in vitro. **A** Conditioned media from wild type MSCs (MSC-WT) and IL-10 engineered MSCs (MSC-IL-10) cultures were serially diluted before treatments, and supernatant collection was performed 24 h after LPS + IFN-γ stimulation for TNF-α quantification by ELISA. Activated macrophages were also treated with dexamethasone (dexa) for comparison. **B** Macrophages and MSC-IL-10 were co-cultured at a 1:1 ratio for 6 h until supernatant collection for TNF-α and IL-10 quantification by ELISA. Controls correspond to unstimulated and LPS + IFN-γ stimulated macrophages. Macrophages were also stimulated (Stim.) with LPS + IFN-γ in the MSC-IL-10 treatments with (+) or without (−) antibodies for IL-10 neutralization (anti-IL-10). Data are presented as mean ± SD analyzed by paired ANOVA with Tukey’s test. ns: not significant. *p < 0.05, **p < 0.005, and ***p < 0.0005

Treatments with the MSCs conditioned media and IL-10-neutralizing antibodies (anti-IL-10) in activated macrophage cultures were performed to confirm if IL-10 secretion by the engineered MSCs was responsible for the enhanced immunomodulatory effects observed in the previous in vitro assays. The increase in TNF-α produced by macrophages stimulated with LPS + IFN-γ was more significantly reduced by the conditioned media from MSC-IL-10 than the conditioned media from MSC-WT in a dilution-dependent manner (Fig. 8A). Moreover, addition of anti-IL-10 reduced IL-10 concentration to basal levels and showed increased TNF-α concentrations in the supernatant of activated macrophages co-cultures with MSC-IL-10, as shown in Fig. 8B. Therefore, despite complete neutralization of IL-10 in the anti-IL-10-treated co-cultures, there was a significant increase in TNF-α compared with the co-culture without anti-IL-10 and a significant reduction compared with the LPS + IFN-γ control, indicating that IL-10 neutralization partially reduced the inhibitory effect exerted by MSC-IL-10 on TNF-α production by activated macrophages.

### Immunomodulation in vivo

**Fig. 9:**
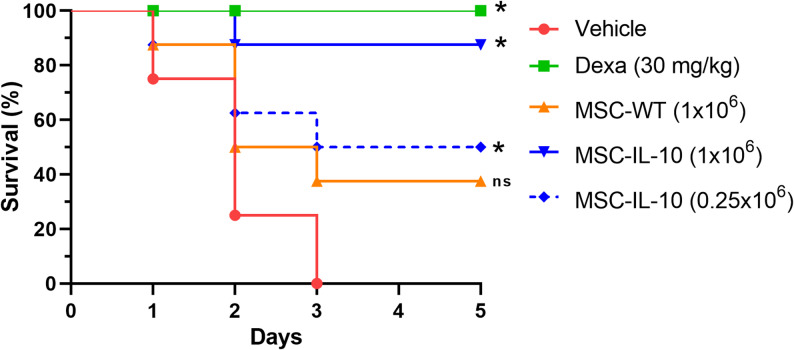
Survival rate of treated mice induced to septic shock. The percentage of mice that survived a lethal dose of LPS is shown for 5 days following treatments with vehicle, dexamethasone, MSC-WT or MSC-IL-10 at doses 0.25 and 1 × 10^6^ cells (*n* = 8). Statistical analysis was performed using the log-rank (Mantel–Cox) test relative to vehicle control. ns: not significant. *p < 0.05

Analyses of protection against septic shock and hematological inflammatory markers were performed in in vivo models of experimental endotoxemia to compare the immunomodulatory effects of the MSCs genetically modified to express IL-10 with non-genetically modified MSCs and standard dexamethasone treatments. In the LPS-induced endotoxic shock model, mice treated with 30 mg/kg of dexamethasone showed the highest survival rate (100%), followed by those treated with 1 × 10⁶ MSC-IL-10 (87.5%), 0.25 × 10⁶ MSC-IL-10 (50%), and 1 × 10⁶ MSC-WT (37.5%) at 5 days after the administration of a lethal dose of LPS. No animals survived over 3 days in the vehicle group (Fig. 9).

**Fig. 10:**
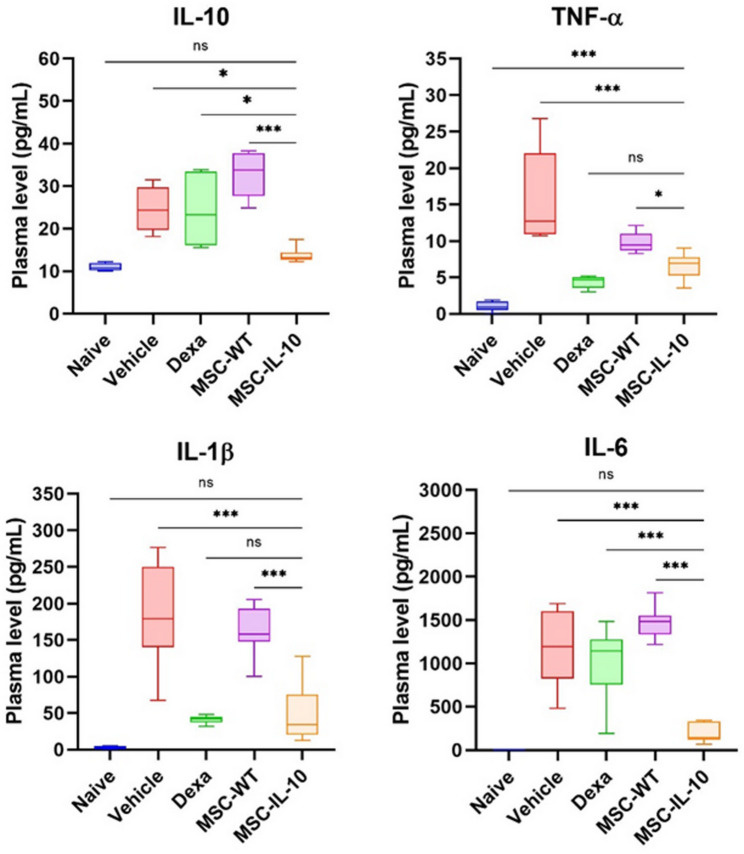
Plasma cytokine concentrations in LPS-challenged mice after treatments. Blood plasma was collected 6–8 h after LPS challenge for quantification of IL-10, TNFα, IL-1β, and IL-6. Groups included naïve animals (healthy, untreated) and LPS-stimulated mice treated with saline (vehicle), dexamethasone (dexa), wild-type MSCs (MSC-WT) or genetically modified cells overexpressing IL-10 (MSC-IL-10) (*n*= 6). Data are presented as mean ± SD analyzed by unpaired one-way ANOVA with Fisher’s LSD tests. ns: not significant. *p < 0.05, **p < 0.005, and ***p < 0.0005

As shown in Fig. 10, quantification of plasma cytokine levels in mice with LPS-induced endotoxemia revealed a marked increase in IL-10, TNFα, IL-1β, and IL-6 in the vehicle group compared to the naïve group. The immunomodulatory activity of MSC-IL-10 in vivo in LPS-challenged mice was also demonstrated by a significant decrease in plasma levels of TNF-α, IL-1β, and IL-6 while IL-10 levels were similar to those in the naïve group. The reduction in plasma levels of these cytokines was significantly greater in the MSC-IL-10 treatment than in the MSC-WT treatment. Cytokine concentrations in the plasma of mice treated with MSC-IL-10 were not statistically different from those of the naïve group for IL-10, IL-1β, and IL-6, whereas they were not statistically different from those of the dexamethasone-treated group for TNF-α and IL-1β. Notably, plasma IL-6 levels were lower in animals treated with MSC-IL-10 than in dexamethasone-treated mice.

**Fig. 11:**
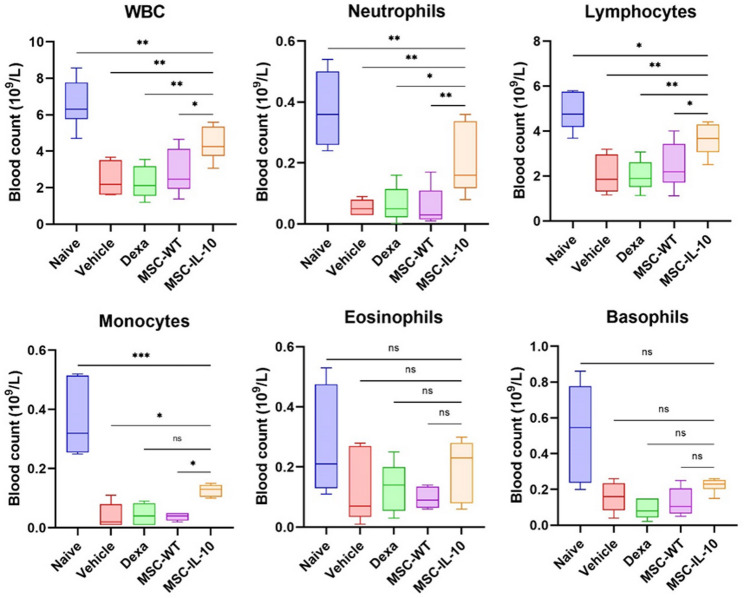
Peripheral blood leukocyte counts in mice with LPS-induced inflammation following treatments. Experimental groups included naïve (healthy, untreated) mice and LPS-challenged mice treated with vehicle, dexamethasone, wild-type mesenchymal stem/stromal cells (MSC-WT), or mesenchymal stem/stromal cells genetically modified to overexpress IL-10 (MSC-IL-10) (*n* = 6). Data are presented as mean ± SD and were analyzed using unpaired one-way ANOVA followed by Fisher’s LSD post hoc test. ns: not significant. *p* < 0.05, *p < 0.005, and **p < 0.0005

Hematological analysis demonstrated that the blood leukocyte count observed in mice of the naïve group was markedly reduced by vehicle-treated, LPS-challenged mice (leukopenia). This condition is characteristic of endotoxemia and results from leukocyte adhesion processes to the endothelium during inflammation, mainly through interactions between integrin receptors on leukocytes and ICAM and VCAM ligands on endothelial cells, leading to leukocyte migration from the bloodstream into inflamed tissues. A significant reduction in leukocyte (WBC) migration to tissues during inflammation was evidenced by a more pronounced increase in the blood counts of these cells, particularly neutrophils, lymphocytes, and monocytes, in the MSC-IL-10 group compared with the vehicle, dexamethasone, and MSC-WT groups. However, the same effect was not observed for eosinophils and basophils (Fig. 11).

**Fig. 12:**
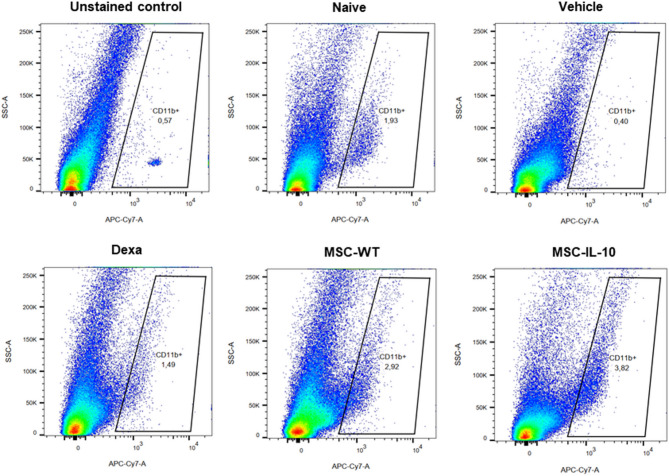
Flow cytometry–based quantification of CD11b⁺ cells in the blood of mice with LPS-induced inflammation following treatments. The percentage of CD11b⁺ cells from the pooled blood samples of each group is shown within the gate corresponding to the low–cell-density area in the unstained control

The reduction in cellular migration from the bloodstream of LPS-challenged mice was confirmed by flow cytometric analysis of CD11b⁺ (Mac-1 integrin) cells in the blood, which are predominantly expressed by activated neutrophils and monocytes. As shown in Fig. 12, the unstained controls and the vehicle group exhibited low percentage of CD11b⁺ cells (0.57% and 0.40%, respectively), whereas the naïve group indicated a basal level of 1.93%. The group of mice treated with 1 × 10⁶ MSC-IL-10 showed 3.82%, representing an 89% increase in CD11b⁺ cells compared with vehicle-treated animals. Increases of 61% and 24% in CD11b⁺ cells were also observed for MSC-IL-10 compared with dexamethasone (1.49%) and MSC-WT (2.92%) treatments, respectively.

### Histological evaluation and biodistribution assay

**Fig. 13:**
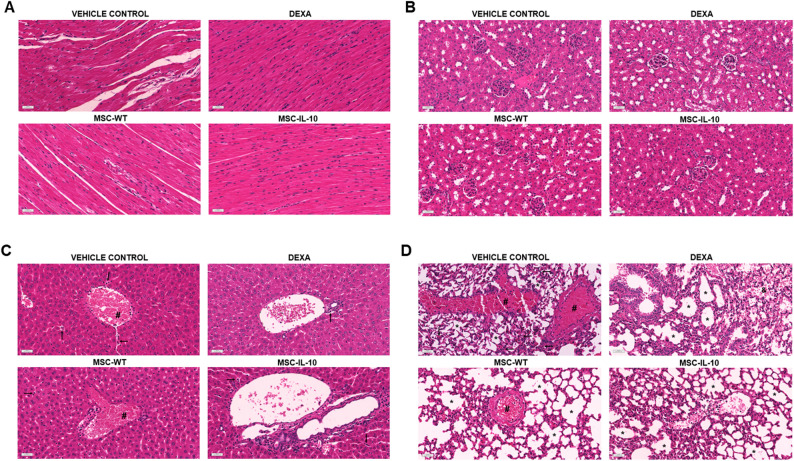
Representative photomicrographs of control and treated animals following LPS challenge. The images illustrate histological analyses of the **A** hearts, **B** kidneys, **C** livers, and **D** lungs of vehicle control mice and animals treated with dexamethasone (dexa), wild type MSCs (MSC-WT) and IL-10 engineered MSCs (MSC-IL-10) after 6–8 h (*n* = 6). #: vascular congestion; arrows: inflammatory infiltrates; *: air spaces; &: hemorrhage

Histological and biodistribution analyses were performed to evaluate if the IL-10-engineered MSCs were able to mitigate inflammatory tissue damage of vital organs while maintaining the typical tropism of MSCs to organs with increased inflammation. Animals in the naïve group (not challenged with LPS) exhibited preserved tissue architecture with no evident histological alterations in hearts, kidneys, liver and lungs (Figure S7). In the animals challenged with LPS from experimental groups, the hearts exhibited preserved tissue architecture, with maintenance of myocardial fiber integrity and absence of inflammatory infiltrate, necrosis, or other significant morphological alterations (Fig. 13A). Renal architecture was also preserved in all experimental groups. Vascular congestion, ranging from moderate to severe, was observed in the cortical and medullary vessels of all animals (Fig. 13B).

The hepatic parenchyma architecture was preserved across all experimental groups challenged with LPS. Vascular congestion, particularly in the portal vein and central venule regions, was observed in all animals, ranging from moderate to severe. Mild inflammatory infiltrates composed of polymorphonuclear and mononuclear cells were noted adjacent to hepatic sinusoids and the portal system. In the negative control group, focal areas of hydropic degeneration and coagulative necrosis of hepatocytes were also identified, indicating localized cellular injury (Fig. 13C).

As illustrated in Fig. 13D, the pulmonary parenchyma architecture of animals challenged with LPS was partially preserved, with thickening of the alveolar septa due to pneumocyte hyperplasia and hypertrophy, in addition to microvascular hyperemia, culminating in areas of atelectasis. Vascular congestion ranged from moderate to severe, accompanied by foci of hemorrhage and edema in the lungs. A predominantly neutrophilic inflammatory infiltrate was identified in focal regions of lung tissue. In the negative control group animals, diffuse alveolar damage was observed, with rupture of the alveolar septa in certain areas. It is noteworthy that inflammatory events were more intense in the negative control group than in the treatment groups, indicating more exacerbated tissue injury.

**Fig. 14:**
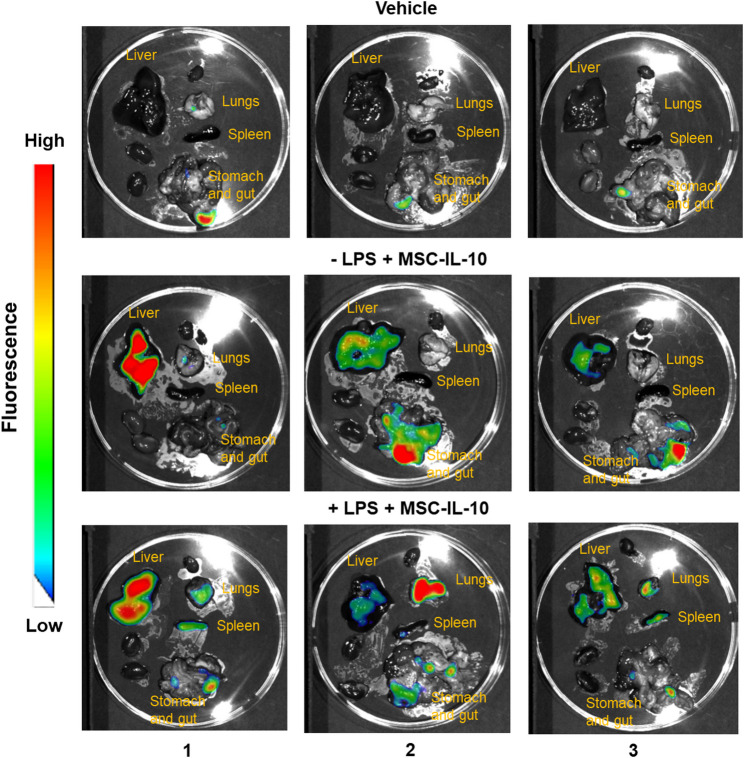
Biodistribution of genetically modified MSCs. Ex vivo biodistribution was demonstrated in triplicate 24 h after i.v. injections of DiR-labeled IL-10-engineered MSCs (MSC-IL-10) by spectral fluorescence imaging. Residual autofluorescence was observed in the stomach of animals in all groups, including the vehicle group. Biodistribution of MSC-IL-10 was observed in the liver of mice that did not receive an LPS dose (− LPS + MSC-IL-10) and animals that received the LPS injection (+ LPS + MSC-IL-10). Localization of MSC-IL-10 in the lungs and spleen was observed only in mice with LPS-induced endotoxemia

As shown in Fig. 14, ex vivo biodistribution of MSC-IL-10 at 24 h exhibited marked differences among organs of mice that received an LPS dose and those that did not. Despite the autofluorescence observed in the stomach of mice in all groups, including the vehicle group, originating from the diet used to feed the animals, medium to high fluorescence was observed in the liver of mice treated with labeled MSC-IL-10 from both groups that received or not an LPS dose. However, in addition to the liver, only mice with LPS-induced endotoxemia and inflammation that received labeled MSC-IL-10 cells exhibited medium to high fluorescence in the lungs and spleen, as demonstrated in the spectral images of these organs.

## Discussion

The use of genetically modified MSCs may represent a promising approach to enhance the therapeutic applications of these cells [7]. MSCs exert immunosuppressive effects through the release of PGE_2_ and TGF-β1, which can promote macrophage polarization toward the M2 phenotype [19, 24]. However, the immunomodulatory efficacy of MSCs still faces limitations, particularly due to variability in the production of immunoregulatory cytokines. By promoting stable overexpression of IL-10, the present study overcomes this limitation by ensuring prolonged and sustained release of an anti-inflammatory cytokine that is not constitutively expressed by MSCs [27]. Németh et al., [19] proposed that bone marrow–derived MSCs reprogram macrophages via PGE_2_ to induce IL-10 production, thereby reducing the severity of sepsis. This study circumvents the indirect production of IL-10 by macrophages and confers a more robust anti-inflammatory potential to MSCs by genetically modifying MSCs to overexpress IL-10.

In this study, constitutive IL-10 expression by MSCs was induced under the EF1α promoter. EF1α-driven expression of transgenes is known for its stability in long-term culture of mammalian cells [35] and it directs transgene expression without silencing during stem cell differentiation [4]. In addition, the use of 1 MOI for lentiviral transduction aimed to reduce cytotoxicity and avoid further genetic alterations that may impair the MSCs’ mesenchymal phenotype, and consequently their safety in prospective clinical applications and inherent therapeutic properties, such as their immunomodulatory and regenerative effects, as well as tropism to inflammatory and damaged tissue sites. The genetically modified MSCs maintained the mesenchymal stromal cell phenotype, as recommended in the guidelines from Renesme et al., [25]. A coding codon sequence optimization of IL-10 was performed to enhance IL-10 expression and secretion without increasing MOI for cell transduction. This strategy was enough to enhance the immunomodulatory capacity of MSCs, as demonstrated by the decreased levels of pro-inflammatory cytokines in vitro and in vivo in the treatments with MSC-IL-10 compared to MSC-WT and dexamethasone controls.

In the in vitro immunomodulatory assays, it was demonstrated that IL-10–expressing MSCs markedly attenuated pro-inflammatory responses, significantly reducing TNF-α, IL-1β, and IL-12 levels with greater efficacy than MSC-WT, an effect consistent with IL-10 secretion and comparable to the dexamethasone treatment. Although no statistically significant downregulation of *Il6* and *Nos2* gene expression relative to control was detected, their expression was more attenuated by MSC-IL-10 than MSC-WT, and this may be explained by the intrinsic expression of these cytokines by MSCs or by their increased expression under co-culture conditions with activated macrophages.

The conditioned medium from MSC-IL-10 induced a greater, dilution-dependent suppression of TNF-α release by macrophages activated with LPS + IFN-γ than that from MSC-WT, suggesting that IL-10 secreted by MSC-IL-10 is likely a key mediator of the enhanced TNF-α reduction. In addition, neutralization of IL-10 partially reversed the suppression of TNF-α in MSC-IL-10 co-cultures, indicating that while IL-10 plays a central role in enhancing the immunomodulatory effects of these cells, additional immunomodulatory factors produced by MSC-IL-10 were also involved in TNF-α reduction.

Consistently, analysis in LPS-challenged mice revealed that MSC-IL-10 treatment significantly reduced systemic inflammation, as evidenced by decreased plasma levels of TNF-α, IL-1β, and IL-6 compared with both vehicle- and MSC-WT-treated groups. Notably, plasma IL-10 levels in MSC-IL-10–treated mice were comparable to those in naïve animals, suggesting reduced and better-resolved inflammation through IL-10 uptake and decreased in vivo production, since IL-10 is upregulated during inflammation to counterbalance its harmful effects. Taken together, these findings indicate that IL-10 overexpression potentiates the immunoregulatory effects of MSCs, resulting in superior suppression of inflammatory mediators both in vitro and in vivo.

As observed in this study, Németh et al., [19] demonstrated that bone marrow–derived MSCs reduced mortality in sepsis induced by cecal ligation and puncture. This effect was also IL-10–dependent, as neutralization of IL-10 abolished the survival benefit. Their MSCs were not genetically modified, but likewise decreased plasma concentrations of pro-inflammatory cytokines such as TNF-α, IL-6, and IL-1β, while increasing IL-10 levels. There was a reduction in lung, liver, and kidney injury, with decreased neutrophilic infiltration and reduced vascular leakage. Treated animals exhibited lower bacterial burden in organs and blood, indicating that MSCs enhance phagocytosis and host defense capacity. MSCs likely secrete PGE_2_, which activates macrophages via EP2/EP4 receptors, leading to IL-10 production and reprogramming of macrophages toward an anti-inflammatory phenotype. Inhibition of PGE_2_ synthesis or EP2 receptor signaling abolished the beneficial effects of MSCs in this model. Here, we indicate that genetically modified IL-10-expressing MSCs further enhance the immunomodulatory effects of MSCs demonstrated by Németh et al. [19].

A meta-analysis of pre-clinical studies confirmed that MSC therapy is safe and improves outcomes in sepsis [33] while glucocorticoid treatment shows limited efficacy and is associated with severe adverse effects in sepsis and septic shock [3]. Therefore, as demonstrated in this study, engineering MSCs to express IL-10 may further enhance the outcomes of MSC therapy in septic shock. A cell therapy with IL-10–expressing MSCs can be more effective in reducing inflammatory responses and may be associated with fewer severe adverse effects.

Howard et al. [12] demonstrated that administration of 1 µg of recombinant IL-10 in mice, 30 min after LPS injection, increased survival from lethal endotoxemia to 90%. In addition, it reduced serum TNF-α concentrations compared with the LPS control for up to 3 h after injection, and this protection was neutralized by administration of anti–IL-10 antibodies. Similarly, the results of the present study are consistent in showing that sustained IL-10 expression by IL-10–expressing MSCs significantly reduced plasma levels of TNF-α, IL-1β, and IL-6 in treated animals and doubled survival (87.5%) in septic shock compared with wild-type MSCs.

Furthermore, the cellular strategy employed in this study may provide greater pharmacokinetic stability, overcoming the short half-life of free recombinant IL-10 demonstrated by Howard et al. [12], who reported a significant reduction in serum TNF-α for only 6 h. Other studies corroborate that IL-10 delivery by MSCs surpasses, in many aspects, the administration of the recombinant protein alone by promoting more sustained local IL-10 levels and prolonged biological effects [14, 29].

The results of this study are consistent with a previous report by Jerkic et al., [13], which demonstrated that both wild-type human umbilical cord–derived MSCs and MSCs genetically modified to overexpress IL-10 reduced TNF-α and IL-6 levels in bronchoalveolar lavage fluid in a rat model of *E. coli*–induced pneumonia. Moreover, they observed increased survival in rats with pneumosepsis treated with IL-10–expressing MSCs (82%) compared with the vehicle group (60%).

Similarly, Wang et al., [36] reported that, in a model of LPS-induced acute lung injury via intratracheal administration, 70% of mice treated on the same day with bone marrow–derived MSCs genetically modified to overexpress IL-10 survived for 96 h, whereas survival in the vehicle group was only 30%. A significant reduction in TNF-α concentration in bronchoalveolar lavage fluid was also observed. In addition, IL-10–expressing MSCs increased the levels of IL-10–producing T and B lymphocytes in the spleen and lungs.

Jerkic et al., [13] further demonstrated that treatment with IL-10–expressing MSCs (but not wild-type MSCs) reduced the number of neutrophils in bronchoalveolar lavage fluid, reinforcing the findings of the present study showing that IL-10–expressing MSCs reduced neutrophil migration from the bloodstream. Additionally, they observed that treatment with IL-10–expressing MSCs increased alveolar space and reduced the fraction of alveolar tissue in the lungs compared with vehicle or wild-type MSCs, indicating that IL-10–expressing MSCs were effective in restoring alveolar architecture, similarly to the histological findings observed in the lungs from this study.

The increased localization of IL-10–expressing MSCs in the lungs of mice with LPS-induced endotoxemia, as demonstrated by the biodistribution assay, is characteristic of these cells, which exhibit tropism toward sites with intense inflammatory activity and tissue injury. While non–LPS-induced animals that received labeled MSC-IL-10 injections maintained the classical physiological biodistribution pattern dominated by the liver, endotoxemic mice likely exhibited increased retention of IL-10–expressing MSCs in the lungs and spleen due to inflammation-induced changes in hemodynamics, vascular permeability, and expression of adhesion molecules in these organs [15].

Untreated sepsis causes drastic leukocyte depletion and tissue infiltration, contributing to immunoparalysis. The greater retention of leukocytes in peripheral blood observed in animals treated with IL-10–expressing MSCs suggests reduced cellular migration to inflamed tissues, which was confirmed by flow cytometric analysis of CD11b⁺ cells. The αMβ2 integrin (CD11b/Mac-1) plays an important role in regulating several immunological functions, including leukocyte adhesion and migration (especially neutrophils, monocytes, and dendritic cells) from the bloodstream through interactions with endothelial ligands ICAM-1 and ICAM-2. The increased retention of CD11b⁺ cells in the blood of mice treated with IL-10–expressing MSCs may be related to a greater effect of IL-10 in reducing activators of the Mac-1 integrin, such as the chemokine CXCL1 and the cytokines TNF-α and IL-1β [8].

MSCs genetically modified to express IL-10 have also demonstrated immunomodulatory effects in other experimental models of inflammation and autoimmune diseases. In a model of rheumatoid arthritis, IL-10–expressing MSCs reduced the clinical severity of the disease, decreased anti-collagen antibody titers, and reduced T-cell proliferative responses, associated with decreased pro-inflammatory cytokine production [6]. In models of uveitis and respiratory allergy, IL-10–expressing MSCs were shown to act through multiple immunological mechanisms, including reduction of inflammatory infiltrates, inhibition of IL-5 and IL-13 production, promotion of macrophage polarization toward a pro-repair phenotype, and increased proportions of regulatory T cells (Tregs), while simultaneously suppressing Th1, Th2, and Th17 responses [14, 17]. MSC-IL-10 also promoted tissue protection and functional recovery in a model of brain injury. Animals transplanted with IL-10–expressing MSCs exhibited improved motor performance and reduced inflammation [22]. In autoimmune myocarditis, IL-10–producing MSCs reduced inflammation and cardiac fibrosis, preserving cardiac function compared with controls treated with unmodified MSCs or recombinant IL-10 [29].

In summary, the results indicate that genetic engineering of MSCs to express IL-10 is a promising strategy to enhance and direct the anti-inflammatory and immunomodulatory activities of MSCs by providing more sustained local IL-10 release, improved regulation of macrophages and T cells, reduced infiltration and tissue damage, and potentially improved functional outcomes in animal models. Additional translational studies are required to evaluate safety, dosage control, duration of expression, and long-term effects before clinical application.

## Conclusions

The present study demonstrates that mesenchymal stem/stromal cells genetically engineered to express IL-10 exhibit enhanced immunomodulatory effects, both in vitro and in vivo, compared to non–genetically modified MSCs. IL-10–expressing MSCs preserved their mesenchymal phenotype and multipotent capacity following genetic modification, and it exerted a more robust suppression of key pro-inflammatory cytokines, including TNF-α, IL-1β, and IL-12 in activated macrophages, as well as TNF-α in activated splenocytes. In a lethal mouse model of LPS-induced endotoxic shock and endotoxemia, treatment with IL-10–expressing MSCs significantly improved survival and reduction of systemic inflammation relative to wild type MSCs, as demonstrated by reduced plasma levels of TNF-α, IL-1β, and IL-6, increased circulating leukocyte counts, decreased migration of CD11b⁺ (Mac-1 integrin) cells from the bloodstream, and attenuated pulmonary tissue injury. These protective effects correlated with preferential biodistribution of IL-10–expressing MSCs to the lungs, the organ most severely affected by LPS-induced inflammation. Collectively, these findings support an enhanced immunomodulatory activity for IL-10-expressing MSCs and highlight the therapeutic promise of combining cell and gene therapy through IL-10 genetic engineering of MSCs as a strategy for the treatment of sepsis and other inflammatory and immune mediated disorders, warranting further translational investigations to evaluate its efficacy and safety.

## Supplementary Information

Below is the link to the electronic supplementary material.

Supplementary Material 1

Supplementary Material 2

## Data Availability

The datasets used and/or analyzed during the current study are available from the corresponding author on reasonable request.

## Abbreviations

CCL2: C-C motif chemokine ligand 2
COX2: Cyclooxygenase 2
FBS: Fetal bovine serum
HO-1: Heme oxygenase-1
IDO: Indoleamine
IFN-γ: Interferon-gamma
IL-10: Interleukin-10
IL-12: Interleukin-12
IL-1β: Interleukin-1 beta
IL-2: Interleukin-2
IL-4: Interleukin-4
IL-6: Interleukin-6
LPS: Lipopolysaccharides
NO: Nitric oxide
NOS2: Nitric oxide synthase 2
PGE_2_: Prostaglandin E2
RT-qPCR: Reverse transcription quantitative polymerase chain reaction
TGF-β: Transforming growth factor-beta
TLR4: Toll like receptor 4
TNF-α: Tumor necrosis factor alpha
