# Supplementary material for: Interleukin-10-engineered mesenchymal stem/stromal cells exhibit robust immunomodulatory effects in vitro and in vivo
Source: Stem Cell Res Ther. 2026 Jun 17;17:274. doi: 10.1186/s13287-026-05093-3 (PMC13459465; doi:10.1186/s13287-026-05093-3)
Supplement: Supplementary file 1 [file 13287_2026_5093_MOESM1_ESM.pdf]

SUPPLEMENTARY MATERIAL

Interleukin-10–engineered mesenchymal stem/stromal cells exhibit robust immunomodulatory effects *in vitro* and *in vivo*

Carneiro, D. C. et al. Stem Cell Research & Therapy, 2026.

Figure S1. Isolation of mesenchymal stem/stromal cells from mouse bone marrow.

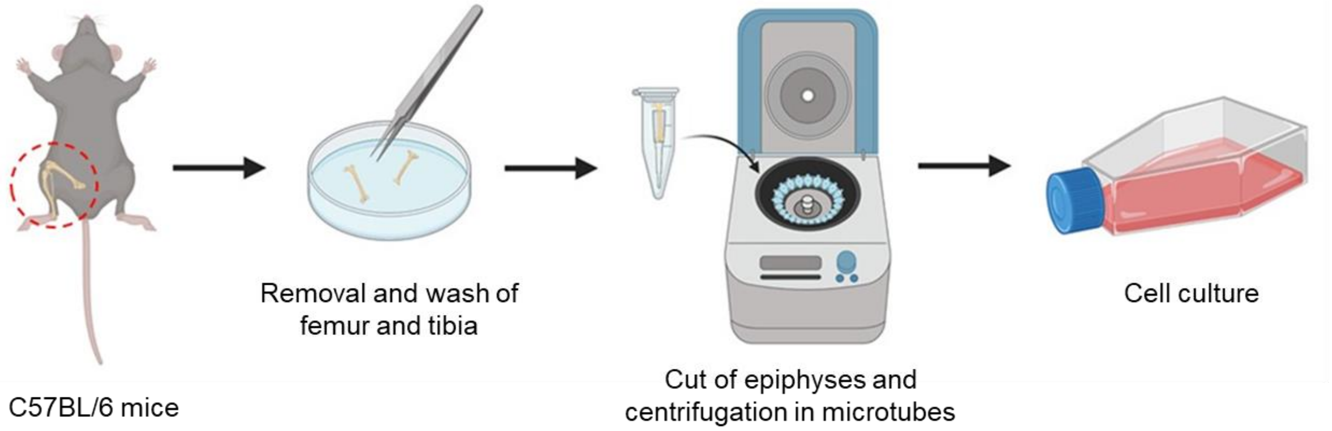

Figure S2. Digestion with BamHI and BsrGI of pEGIP and pMA-RQ with synthetic IL-10.

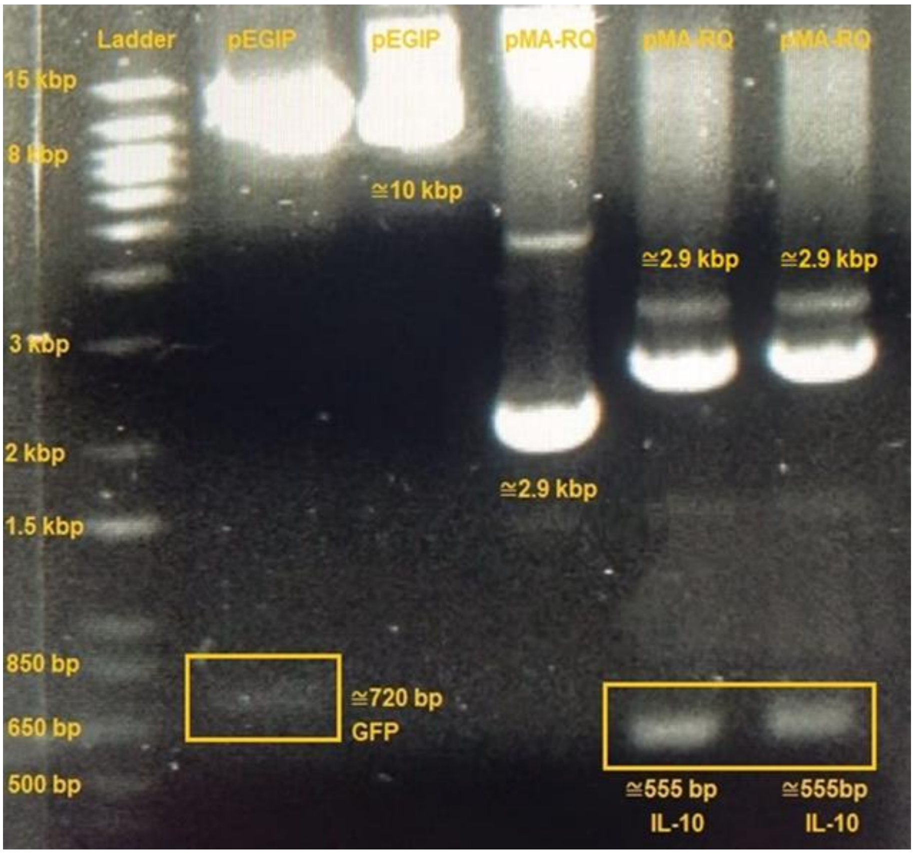

Figure S3. Digestion with BamHI and BsrGI of pEGIP-IL-10 to confirm IL-10 insert.

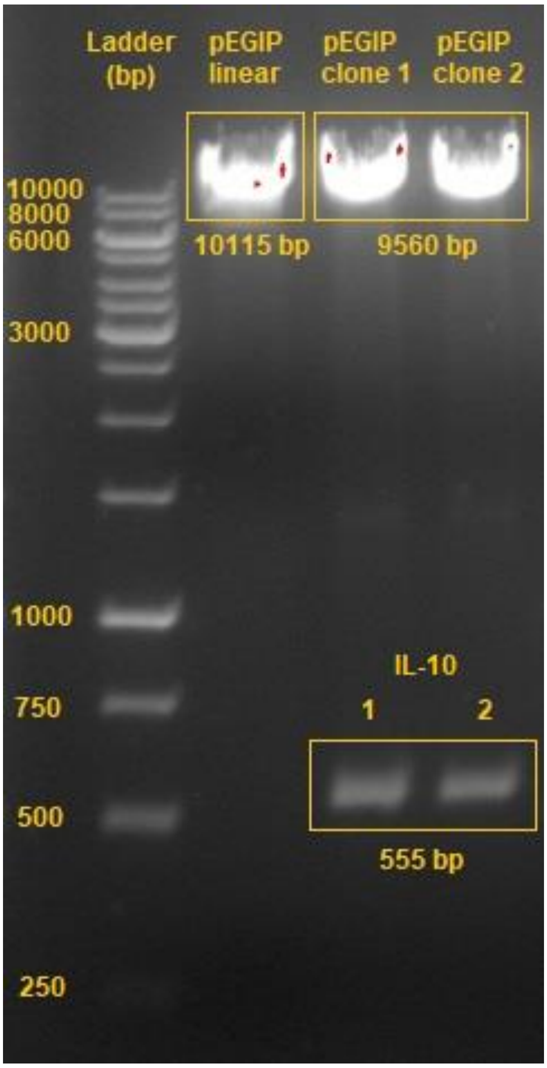

Figure S4. ClustalW multiple alignment between the sequence obtained from sequencing and the predicted sequence for pEGIP-IL-10 at the beginning and end.

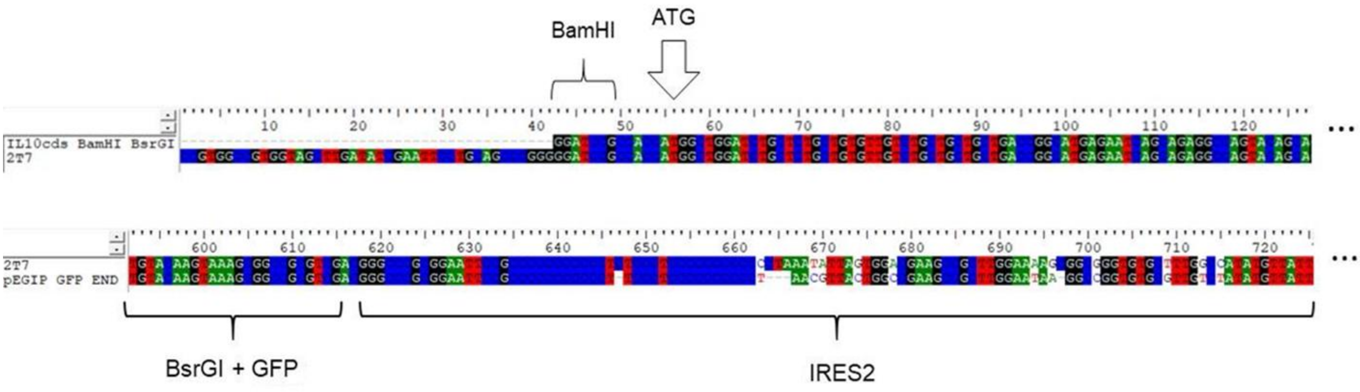

Figure S5. GFP expression by pEGIP and pEGIP-IL-10 transfected HEK293T cells and transduced mesenchymal stem/stromal cells.

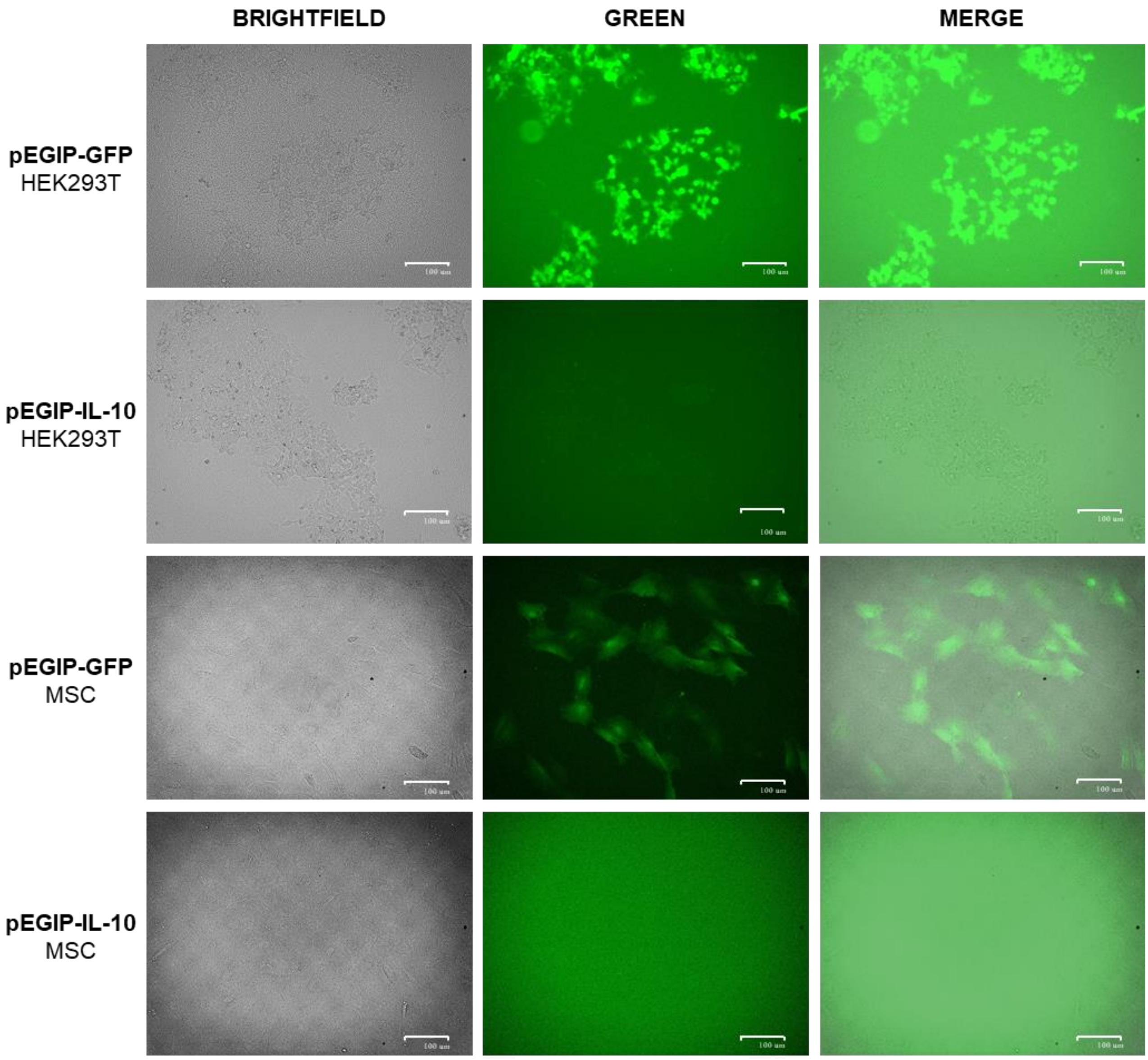

Figure S6. Genetic modification of the mesenchymal stem/stromal cells.

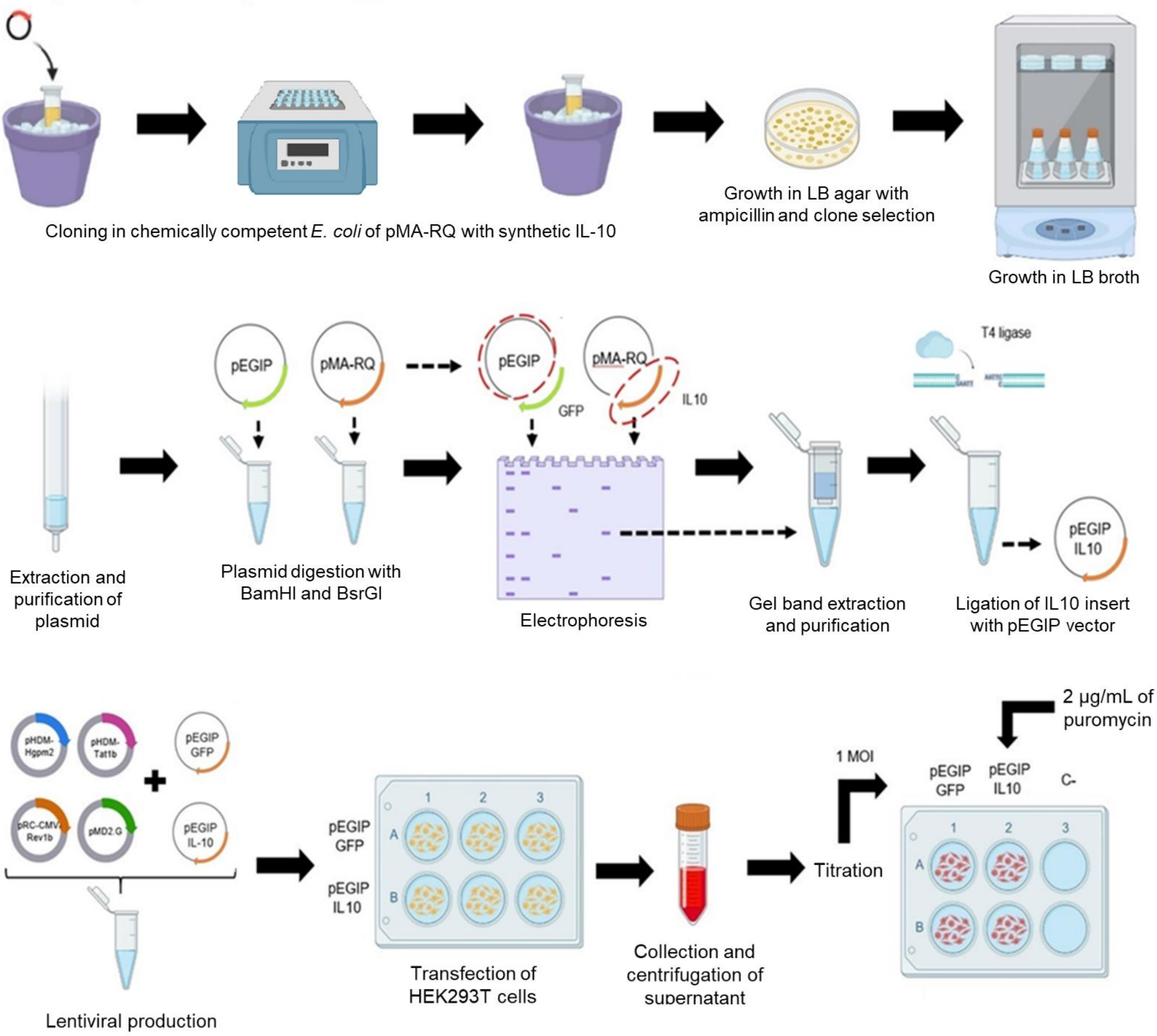

Figure S7. Histological analysis of organs from naïve mice.

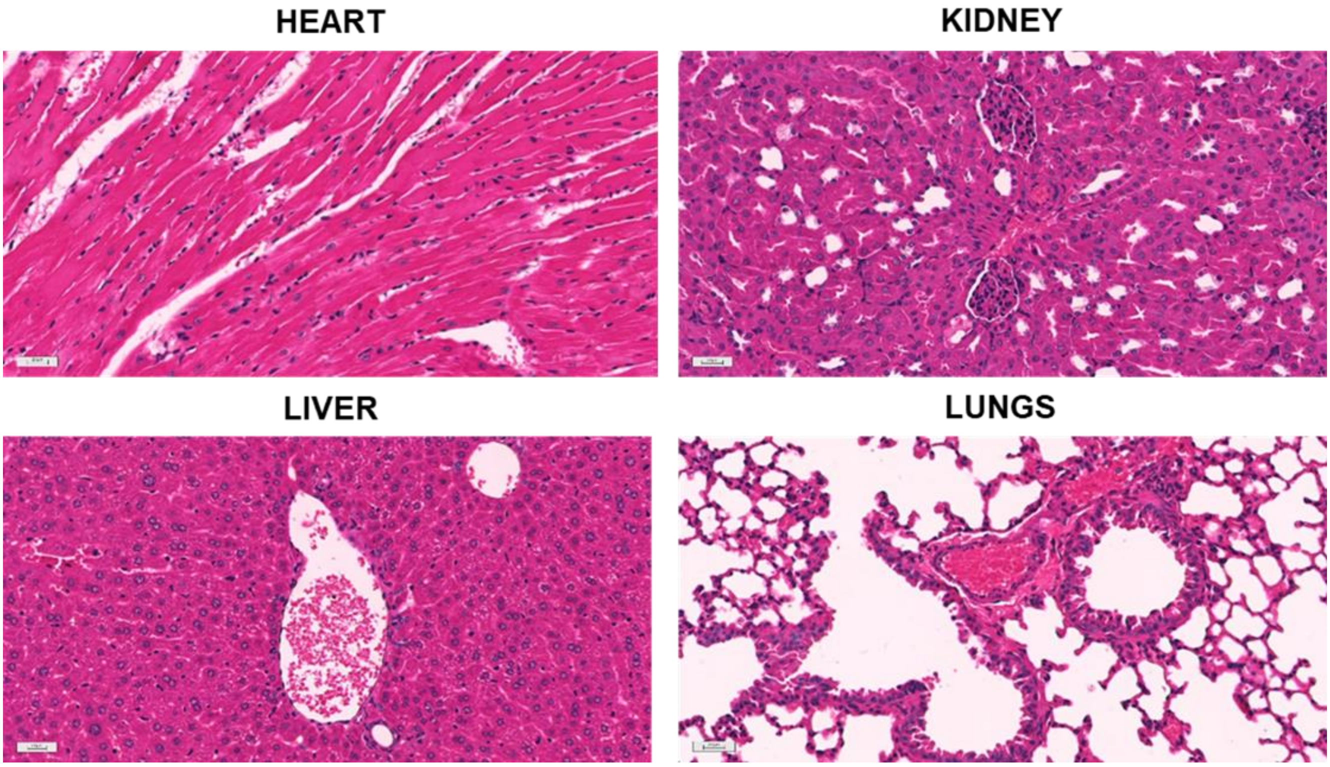

Table S1. Plasmids used in the lentiviral system.

Plasmid	Addgene	Quantity per well
pHDM-Hgpm2	#164441	0,22 µg
pHDM-Tat1b	#164442	0,22 µg
pRC-CMV-Rev1b	#164443	0,22 µg
pMD2.G	#12259	0,34 µg
pEGIP (GFP/IL-10)	#26777	1 µg

Table S2. Target genes and primers used for RT-qPCR.

Target genes	5' to 3' primer sequence	
	Forward	Reverse
Ipo8	CTGAAGGGCACTATCGACCC	TAGGACTGGTTAAGCTCCGTC
Gapdh	AGGTCGGTGTGAACGGATTTG	TGTAGACCATGTAGTTGAGGTCA
Oct4	AGAGGATCACCTTGGGGTACA	CGAAGCGACAGATGGTGGTC
Nanog	TCTTCCTGGTCCCCACAGTTT	GCAAGAATAGTTCTCGGGATGAA
Sox2	GCGGAGTGGAAACTTTTGTCC	CGGGAAGCGTGTACTTATCCTT
Tgfb1	ACTGGAGTTGTACGGCAGTG	GGGGCTGATCCCGTTGATTT
Ifng	ATGAACGCTACACACTGCATC	CCATCCTTTTGCCAGTTCCTC
Cox2	TTCAACACACTCTATCACTGGC	AGAAGCGTTTGCGGTACTCAT
Ccl2	TTAAAAACCTGGATCGGAACCAA	GCATTAGCTTCAGATTTACGGGT
Ido1	GCTTTGCTCTACCACATCCAC	CAGGCGCTGTAACCTGTGT
Ido2	TGGGGAGATACCACATTTCTGA	TGAGGAAGTCTGAGGGCAATTT
Ho1	AAGCCGAGAATGCTGAGTTCA	GCCGTGTAGATATGGTACAAGGA
Il4	CCCCCAGCTAGTTGTCATCC	AGGACGTTTGGCACATCCAT
Il10 (non-optimized)	GCTCTTACTGACTGGCATGAG	CGCAGCTCTAGGAGCATGTG
Il10 (optimized)	CAGATTTCTGCCCTGCGAGA	GTCGAACTCGTTCATGGCCT
Tnfa	GTAGCCCACGTCGTAGCAA	ACAAGGTACAACCCATCGGC
Il1b	TGCCACCTTTTGACAGTGATG	AAGGTCCACGGGAAAGACAC
Il6	CCCCAATTTCCAATGCTCTCC	CGCACTAGGTTTGCCGAGTA
Il12	AGACATCACACGGGACCAAAC	CCAGGCAACTCTCGTTCTTGT

Table S3. Percentage of cells expressing positive and negative mesenchymal markers from the cultured wild type MSCs (MSC-WT) and IL-10 engineered MSCs (MSC-IL-10).

Marker	Type	MSC-WT (%)	MSC-IL-10 (%)
CD105	Positive	92.3 ± 4.0	94.3 ± 1.1
CD73		81.3 ± 1.7	95.0 ± 2.5
CD29		95.4 ± 0.2	93.2 ± 0.5
CD45	Negative	4.6 ± 2.1	1.95 ± 1.5
CD34		27.7 ± 2.9	17.1 ± 1.5
CD11b		1.4 ± 0.4	0.81 ± 0.9
