# Supplementary material for: Interleukin-10-engineered mesenchymal stem/stromal cells exhibit robust immunomodulatory effects in vitro and in vivo
Source: Stem Cell Res Ther. 2026 Jun 17;17:274. doi: 10.1186/s13287-026-05093-3 (PMC13459465; doi:10.1186/s13287-026-05093-3)
Supplement: Supplementary file 2 [file 13287_2026_5093_MOESM2_ESM.pdf]

NOTE: Please save this file locally before filling in the table, DO NOT work on the file within your internet browser as changes will not be saved. Adobe Acrobat Reader (available free here) is recommended for completion.

The ARRIVE guidelines 2.0: author checklist

The ARRIVE Essential 10

These items are the basic minimum to include in a manuscript. Without this information, readers and reviewers cannot assess the reliability of the findings.

Item		Recommendation	Section/line number, or reason for not reporting
**Study design**	1	For each experiment, provide brief details of study design including:<br>a. The groups being compared, including control groups. If no control group has been used, the rationale should be stated.<br>b. The experimental unit (e.g. a single animal, litter, or cage of animals).	Naive, i.v. injections of vehicle, 1x10^6 MSC-WT, 0.25 or 1x10^6 MSC-IL-10, and i.p. injections of dexamethasone (30 mg/kg) 30 min after challenge with i.p. injections of 600 µg of LPS.<br>Single animal.
**Sample size**	2	a. Specify the exact number of experimental units allocated to each group, and the total number in each experiment. Also indicate the total number of animals used.<br>b. Explain how the sample size was decided. Provide details of any *a priori* sample size calculation, if done.	8/group for endotoxic shock model and 6/group for blood and histological analyses. Respectively, 40, 30 and 30 total number in each experiment. 140 total number used.<br>Decided as previously described by Meira et al. (2017).
**Inclusion and exclusion criteria**	3	a. Describe any criteria used for including and excluding animals (or experimental units) during the experiment, and data points during the analysis. Specify if these criteria were established *a priori*. If no criteria were set, state this explicitly.<br>b. For each experimental group, report any animals, experimental units or data points not included in the analysis and explain why. If there were no exclusions, state so.<br>c. For each analysis, report the exact value of *n* in each experimental group.	A priori: male BALB/c mice with 14-18 g of weight for endotoxic shock model or 14-16 weeks of age for blood and histological analyses.<br>No exclusions.<br>n=8/group for endotoxic shock model and n=6/group for blood and histological analyses.
**Randomisation**	4	a. State whether randomisation was used to allocate experimental units to control and treatment groups. If done, provide the method used to generate the randomisation sequence.<br>b. Describe the strategy used to minimise potential confounders such as the order of treatments and measurements, or animal/cage location. If confounders were not controlled, state this explicitly.	Simple randomisation was used to allocate experimental units.<br>All experimental procedures and assessments were performed at consistent times of day using standardized protocols, reagents, animal allocation, and ballanced groups.
**Blinding**	5	Describe who was aware of the group allocation at the different stages of the experiment (during the allocation, the conduct of the experiment, the outcome assessment, and the data analysis).	No blinding.
**Outcome measures**	6	a. Clearly define all outcome measures assessed (e.g. cell death, molecular markers, or behavioural changes).<br>b. For hypothesis-testing studies, specify the primary outcome measure, i.e. the outcome measure that was used to determine the sample size.	Survival, plasma cytokine quantification, leukocyte counts, flow cytometry of CD11b+ cells and histological analyses.<br>Survival.
**Statistical methods**	7	a. Provide details of the statistical methods used for each analysis, including software used.<br>b. Describe any methods used to assess whether the data met the assumptions of the statistical approach, and what was done if the assumptions were not met.	Mean and standard deviation analyses of data using GraphPad Prism 8 by log-rank (Mantel–Cox) test and ANOVA with Fisher's LSD tests.<br>Parametric analyses were performed when assumptions were satisfied. The assumptions were not violated.
**Experimental animals**	8	a. Provide species-appropriate details of the animals used, including species, strain and substrain, sex, age or developmental stage, and, if relevant, weight.<br>b. Provide further relevant information on the provenance of animals, health/immune status, genetic modification status, genotype, and any previous procedures.	Male BALB/c mice with 14-18 g of weight for endotoxic shock model or 14-16 weeks of age for blood and histological analyses.<br>Animals were sourced from accredited specific pathogen–free (SPF) facility. They were clinically healthy at study initiation and immunologically naïve, and had not undergone prior experimental procedures
**Experimental procedures**	9	For each experimental group, including controls, describe the procedures in enough detail to allow others to replicate them, including:<br>a. What was done, how it was done and what was used.<br>b. When and how often.<br>c. Where (including detail of any acclimatisation periods).<br>d. Why (provide rationale for procedures).	Randomization of five experimental groups of male BALB/c mice with 14-18 g of weight for endotoxic shock model or 14-16 weeks of age for blood and histological analyses.<br>Twice. Monitoring for five days for endotoxic shock model. Collection of blood and organ samples 6-8 h after LPS challenge. Execution of procedures in specific<br>To evaluate protection against septic shock and analyze hematological inflammatory markers in in vivo models of experimental endotoxemia, and to compare the
**Results**	10	For each experiment conducted, including independent replications, report:<br>a. Summary/descriptive statistics for each experimental group, with a measure of variability where applicable (e.g. mean and SD, or median and range).<br>b. If applicable, the effect size with a confidence interval.	Mean and SD.<br>Interquartile ranges for effect size and confidence interval interpretation are reported in box plots.
